# Analysis of Structural Genomic Diversity in *Aegilops umbellulata*, *Ae. markgrafii*, *Ae. comosa*, and *Ae. uniaristata* by Fluorescence *In Situ* Hybridization Karyotyping

**DOI:** 10.3389/fpls.2020.00710

**Published:** 2020-06-09

**Authors:** Zhongping Song, Shoufen Dai, Tingyu Bao, Yuanyuan Zuo, Qin Xiang, Jian Li, Gang Liu, Zehong Yan

**Affiliations:** ^1^State Key Laboratory of Crop Gene Exploration and Utilization in Southwest China, Wenjiang, China; ^2^Triticeae Research Institute, Sichuan Agricultural University, Wenjiang, China

**Keywords:** fluorescence *in situ* hybridization (FISH), chromosomal variation, karyotypes, diploid *Aegilops* species, *Ae. umbellulata*, *Ae. markgrafii*, *Ae. comosa*, *Ae. uniaristata*

## Abstract

Fluorescence *in situ* hybridization karyotypes have been widely used for evolutionary analysis on chromosome organization and genetic/genomic diversity in the wheat alliance (tribe Triticeae of Poaceae). The karyotpic diversity of *Aegilops umbellulata*, *Ae. markgrafii*, *Ae. comosa* subsp. *comosa* and subsp. *subventricosa*, and *Ae. uniaristata* was evaluated by the fluorescence *in situ* hybridization (FISH) probes oligo-pSc119.2 and pTa71 in combination with (AAC)_5_, (ACT)_7_, and (CTT)_12_, respectively. Abundant intra- and interspecific genetic variation was discovered in *Ae. umbellulata*, *Ae. markgrafii*, and *Ae. comosa*, but not *Ae. uniaristata.* Chromosome 7 of *Ae. umbellulata* had more variants (six variants) than the other six U chromosomes (2–3 variants) as revealed by probes oligo-pSc119.2 and (AAC)_5_. Intraspecific variation in *Ae. markgrafii* and *Ae. comosa* was revealed by oligo-pSc119.2 in combination with (ACT)_7_ and (CTT)_12_, respectively. At least five variants were found in every chromosome of *Ae. markgrafii* and *Ae. comosa*, and up to 18, 10, and 15 variants were identified for chromosomes 2 of *Ae. markgrafii*, 4 of *Ae. comosa* subsp. *comosa*, and 6 of *Ae. comosa* subsp. *subventricosa*. The six *Ae. uniaristata* accessions showed identical FISH signal patterns. A large number of intra-specific polymorphic FISH signals were observed between the homologous chromosomes of *Ae. markgrafii* and *Ae. comosa*, especially chromosomes 1, 2, 4, and 7 of *Ae. markgrafii*, chromosome 4 of *Ae. comosa* subsp. *comosa*, and chromosome 6 of *Ae. comosa* subsp. *subventricosa*. Twelve *Ae. comosa* and 24 *Ae. markgrafii* accessions showed heteromorphism between homologous chromosomes. Additionally, a translocation between the short arms of chromosomes 1 and 7 of *Ae. comosa* PI 551038 was identified. The FISH karyotypes can be used to clearly identify the chromosome variations of each chromosome in these *Aegilops* species and also provide valuable information for understanding the evolutionary relationships and structural genomic variation among *Aegilops* species.

## Introduction

The genus *Aegilops* serves as a valuable genetic resource for expanding the genetic basis of cultivated bread wheat, as it is closely related to *Triticum* and has played a pivotal role in the evolution of bread wheat (Schneider et al., 2008). *Aegilops* contains 11 diploid species that harbor different nuclear and cytoplasmic genomes (Van Slageren, 1994). The diploid *Aegilops* species include seven basic genomes, namely U, C, M, N, D, S, and T (Kimber and Tsunewaki, 1988). *Ae. umbellulata*, *Ae. markgrafii*, *Ae. comosa*, and *Ae. uniaristata* are diploid donor species of polyploid *Aegilops* harboring the U, C, M, and N genomes, respectively.

In addition to the evolutionary contribution to polyploid *Aegilops* species, the diploid *Aegilops* species including those with U, M, C, and N genomes also harbor abundant beneficial genes for the genetic improvement of bread wheat with respect to, for instance, resistance to leaf and stripe rust (Sears, 1956; Riley et al., 1968; Riar et al., 2012; Toor et al., 2016; Bansal et al., 2017; Liu et al., 2019) and powdery mildew (Gill et al., 1985; Zhu et al., 2006; Weidner et al., 2012), tolerance to salt (Gorham, 1990) and aluminum stress (Miller et al., 1995), accumulation of zinc and iron (Wang et al., 2011; Neelam et al., 2012), high efficiency in zinc uptake (Cakmak et al., 1999), as well as high protein content and gluten content (Gong et al., 2017; Wang et al., 2017).

*Aegilops* species are distributed from the Mediterranean via Southwest Asia to central Asia and contain sufficient genetic diversity for adaptation to various environments. Several methods, including morphological observation (Kawahara, 2002; Tahernezhad et al., 2010), C-banding karyotype (Badaeva et al., 1996a), biochemical markers (Rodríguez-Quijano et al., 2001; Dai et al., 2015), and molecular markers (Sasanuma et al., 2004; Tahernezhad et al., 2010; Thomas and Bebeli, 2010), have been adopted to assess the genetic diversity and evolutionary relationships of *Aegilops* species. The karyotypes of some *Aegilop*s species have been established with a C-banding technique (Teoh et al., 1983; Friebe et al., 1992, 1995, 1996b). Hybridization signals of the (CTT)_n_ probe on *Aegilops* chromosomes are often consistent with their C-banding patterns (Ruban and Badaeva, 2018). Furthermore, some cloned repeats and oligo-nucleotide sequences have been used as probes to establish fluorescence *in situ* hybridization (FISH) karyotypes rather than C-banding karyotypes due to improved efficiency and easier operation.

Fluorescence *in situ* hybridization is a valid tool for the direct physical mapping of DNA sequences on chromosomes and is often utilized in evolutionary and speciation studies as well as for the assessment of genetic diversity among and within species (Badaeva et al., 2002, 2004, 2015). Several probes, such as pSc119 or oligo-pSc119.2, Afa family, (AAC)_5_, (GAA)_n_, oligo-pTa535, and oligo-pTa71, have been used for the FISH karyotyping of diploid *Aegilops* species, including *Ae. umbellulata*, *Ae. markgrafii*, *Ae. comosa*, and *Ae. uniaristata*, and their introgression lines with wheat (Badaeva et al., 1996a, 2011; Iqbal et al., 2000; Molnár et al., 2011, 2015, 2016; Kwiatek et al., 2013; Mirzaghaderi et al., 2014; Danilova et al., 2017; Liu et al., 2019; Song et al., 2019).

The goatgrasses, including *Ae. umbellulata*, *Ae. markgrafii*, *Ae. comosa*, and *Ae. uniaristata*, are necessary germplasms for the genetic improvement of cultivated hexaploid wheat and exhibit rich genetic diversity at the chromosome level. In previous studies, the FISH karyotypes were mainly used for chromosome identification and, to a lesser extent, for the analysis of genomic diversity, chromosome organization, and evolutionary patterns. Currently, FISH karyotype-based chromosome organization and the evolutionary patterns of these *Aegilops* species are still insufficiently. Therefore, the main objectives of the present study were to elucidate the genetic diversity of the four diploid *Aegilops* species using FISH karyotypes and to understand their chromosomal organization. In the present investigation, six FISH probes were tested for the selection of suitable probes for evaluating the diversity of four diploid *Aegilops* species with U, C, M, and N chromosomes. These data provide useful information for understanding the genome evolution and differentiation as well as the genetic diversity of *Aegilops* species.

## Materials and Methods

### Plant Materials

A collection of 145 accessions belonging to *Ae. umbellulata* (2*n* = 2*x* = 14, UU, 47 accessions), *Ae. markgrafii* (2*n* = 2*x* = 14, CC, 44 accessions), *Ae. comosa* (2*n* = 2*x* = 14, MM, 13 accessions of subsp. *comosa* and 35 accessions of subsp. *subventricosa*), and *Ae. uniaristata* (2*n* = 2*x* = 14, NN, six accessions) were subjected to FISH karyotyping (Table 1). These materials were supplied from the USDA-ARS germplasm bank^1^.

**TABLE 1:** Materials and chromosome types.

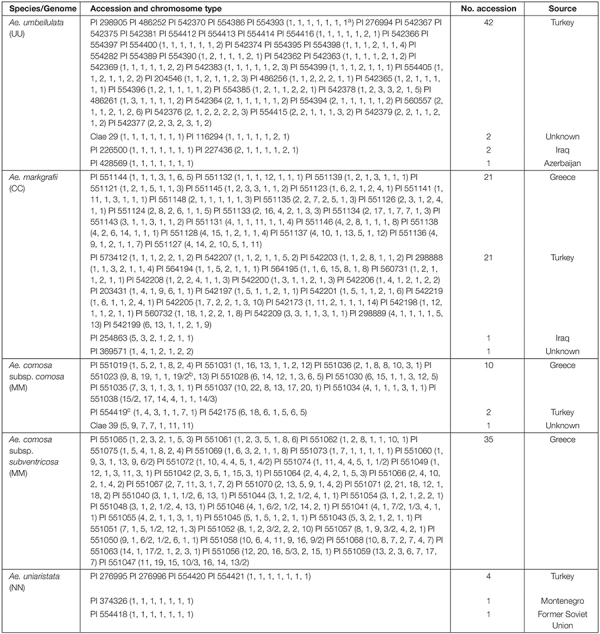

*a. Represent combinations of 1-7 homologous chromosomes listed in Figures 2, 3. b. In Ae. comosa, the symbols 19/2 means chromosome combinations of probes oligo-pSc119.2 and (CTT)_12_, and oligo-pTa71. c. Previously classified as Ae. uniaristata, our analysis verified that it should be Ae. comosa.*

### FISH Karyotyping

Ten randomly selected seeds from each accession were germinated on Petri dishes lined with double-layer moist filter papers at 4°C for ∼24 h and then incubated in a container under a 16 h photoperiod (light/dark temperature 22/16°C). Root tips were excised when the roots reached 1–2 cm and were treated with 1.0 MPa nitrous oxide (NO) gas for 2 h. Then, the root tips were fixed in glacial acetic acid for at least 5 min before storing in 70% ethanol for slide preparation (Kato, 1999). Root tips were suspended in cellulase/pectinase enzyme solution (4: 2) before dropping onto slides (Komuro et al., 2013). The FISH procedure was the same as described by Hao et al. (2013). Ten microliters of hybridization mixture solution was added to each slide. Each slide was then placed at 37°C for at least 1 h. DAPI (4′, 6-diamidino-2-phenylindole) was used to counter-stain the slides for visualizing the FISH signals. The chromosomal observations of hybridization signals were conducted with an Olympus BX-63 epifluorescence microscope, and the photographs were recorded with a Photometric SenSys Olympus DP80 CCD camera (Olympus, Tokyo, Japan). After capturing the images, the coverslips of each slide were removed and the slides were washed for the next FISH (Komuro et al., 2013). Original images were processed using Photoshop V7.0 (Adobe Systems Incorporated, United States). At least five metaphase cells of each accession were observed in order to count chromosome variants.

### DNA Probes and Chromosome Identification

Six probes, namely oligo-pSc119.2, oligo-pTa71, oligo-pTa713, (AAC)_5_, (CTT)_12_, and (ACT)_7_, which are suited for use in identifying the chromosomes of common wheat and *Aegilops* species (Cuadrado and Jouve, 2010; Tang et al., 2014; Zhao et al., 2016) were used in the current investigation. The chromosomes of the four diploid *Aegilops* species were identified and classified based on the FISH patterns of these DNA probes combined with the C-banding karyotypes in previous investigations (Friebe et al., 1995, 1996a; Badaeva et al., 1996a; Danilova et al., 2017; Liu et al., 2019; Song et al., 2019). The 5′-ends of oligo-pSc119.2 and oligo-pTa71 were labeled with 6-carboxyfluorescein (6-FAM), and the remaining four probes were labeled with 6-carboxy tetramethylrhodamine (Tamra). All of these probes were synthesized by Sangon Biotech (Shanghai, China).

## Results

### FISH Markers for Each Species

**FIGURE 1:**
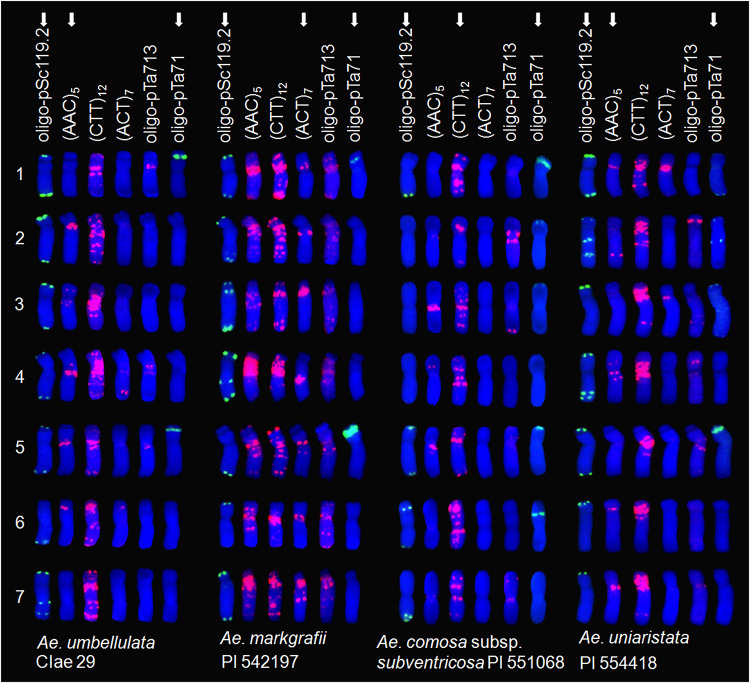
FISH karyotyping of the chromosomes of four diploid *Aegilops* species using six DNA probes. The probes eventually used for polymorphism analysis of each diploid *Aegilops* species are shown by white arrowheads (see text for details).

Six probes were initially screened for one accession each from four diploid species, namely *Ae. umbellulata* (accession CIae 29), *Ae. markgrafii* (PI 542197), *Ae. comosa* subsp. *subventricosa* (PI 551068), and *Ae. uniaristata* (PI 554418) (Figure 1).

Three [oligo-pSc119.2, (AAC)_5_ and (CTT)_12_] of the six probes showed hybridization signals on nearly all U chromosomes of *Ae. umbellulata*, while the remaining three probes had hybridization signals on some U chromosomes, including 1U, 4U, and 5U. The (CTT)_12_ probe hybridized to many sites on every U chromosome and stretched throughout the whole chromosome, whereas (ACT)_7_ and oligo-pTa713 harbored a few signals on the pericentromeric region of chromosomes 1U, 4U, and 5U and chromosome arms 4UL and 6US. The probes oligo-pSc119.2 and (AAC)_5_ hybridized to the telomeric regions of every U chromosome and the pericentromeric regions of chromosomes 2U, 3U, 4U, 5U, and 6U, and their combinations showed hybridization signals on every U chromosome and could distinguish 1U, 3U, 4U, 6U, and 7U. Although oligo-pTa71 only hybridized to the nucleolar organizing regions of chromosome arms 1US and 5US, it was helpful to differentiate chromosomes 1U and 5U from 2U, 3U, and 4U.

Five of the six probes (all except for oligo-pTa71) hybridized to all C chromosomes of *Ae. markgrafii*. The oligo-pSc119.2 probe hybridized to the telomeric regions of chromosomes 1C, 2C, 3C, 4C, and 7C and chromosome arms 6CS and 5CL, as well as the central region of chromosome arm 4CL. The hybridization sites of probes (AAC)_5_, (ACT)_7_, (CTT)_12_, and oligo-pTa713 were found on every C chromosome, while those of oligo-pTa71 hybridized to the nucleolar organizing regions of chromosome arms 1CS and 5CS. The probe combinations oligo-pSc119.2/(ACT)_7_ and oligo-pTa71 could clearly differentiate all C chromosomes.

All six probes other than (ACT)_7_ harbored hybridization sites on the M chromosomes of *Ae. comosa*. The hybridization sites of the oligo-pTa713 probe were located on the telomeric region of chromosome arm 3ML, near the telomeric region of chromosome arm 7MS, and the pericentromeric regions of chromosomes 2M and 7M, while those of (AAC)_5_ were located on the pericentromeric regions of chromosomes 4M to 7M. The oligo-pSc119.2 probe hybridized to the telomeric region of chromosome arms 1ML and 7ML and chromosomes 5M and 6M. However, the hybridization sites (CTT)_12_ mainly targeted the pericentromeric region and telomeric region of chromosomes 1M to 7M. The oligo-pTa71 probe hybridized to sites on chromosome arm 1MS and chromosomes 2M to 6M. The probes oligo-pSc119.2/(CTT)_12_ and oligo-pTa71 had the ability to distinguish each M chromosome. The remaining three probes, (AAC)_5_, (ACT)_7_, and oligo-pTa713, were not further used as they lacked sufficient hybridization sites on the M chromosome.

All six probes hybridized to sites on the N chromosomes of *Ae. uniaristata.* The hybridization signals of the (AAC)_5_ probe were distributed on the pericentromeric regions of chromosomes 1N, 4N, 6N, and 7N and chromosome arm 2NL. The oligo-pSc119.2 hybridized to the telomeric regions on chromosome arms 2NS, 3NS, 6NS, 7NS, and chromosomes 1N, 4N, and 5N, as well as the central region on chromosome arm 2NL. The hybridization signal sites (CTT)_12_ were mainly on the pericentromeric regions of every N chromosome, while those of (ACT)_7_ were on the pericentromeric regions of chromosomes 1N and 3N. The oligo-pTa713 hybridized to pericentromeric regions on chromosomes 4N, 5N, and 7N and near the telomeric region on chromosome arm 2NS and the middle region of chromosome arm 3NL. The oligo-pTa71 probe exhibited strong hybridization signals on nucleolar organizing regions 5NS and weak signals on the pericentromeric regions of chromosomes 1N and 2N and the telomeric regions of chromosome arms 1NL and 2NS and chromosome 3N. The probes oligo-pSc119.2/(AAC)_5_ could clearly differentiate every N chromosome with the aid of oligo-pTa71.

Among the selected accession in each species, the oligo-pSc119.2 probe showed strong signals on all seven chromosomes of *Ae. umbellulata*, *Ae. markgrafii*, and *Ae. uniaristata*, as well as on the four chromosomes (1M, 5M, 6M, and 7M) of *Ae. comosa*. The (AAC)_5_ signals were distributed on every chromosome of *Ae. markgrafii*, on five chromosomes of *Ae. umbellulata* (2–6U) and *Ae. uniaristata* (1N, 2N, 4N, 6N, and 7N), and on six chromosomes (except for 1M) of *Ae. comosa*. The (ACT)_7_ signals of *Ae. markgrafii* were presented on all seven chromosomes, while only a few or no such signal was detected from the other three *Aegilops* species. Of the six probes, (CTT)_12_ had the most signal points, while oligo-pTa713 and oligo-pTa71 exhibited the fewest signal points. The (CTT)_12_ probe showed strong signals on all of the seven chromosomes of the four *Aegilops* species, but the signals were mainly concentrated on the pericentromeric regions. There were more (CTT)_12_ signals in *Ae. umbellulata* and *Ae. markgrafii* than in *Ae. comosa* and *Ae. uniaristata*.

### Polymorphic Variants for Each Species

#### Ae. umbellulata

**FIGURE 2:**
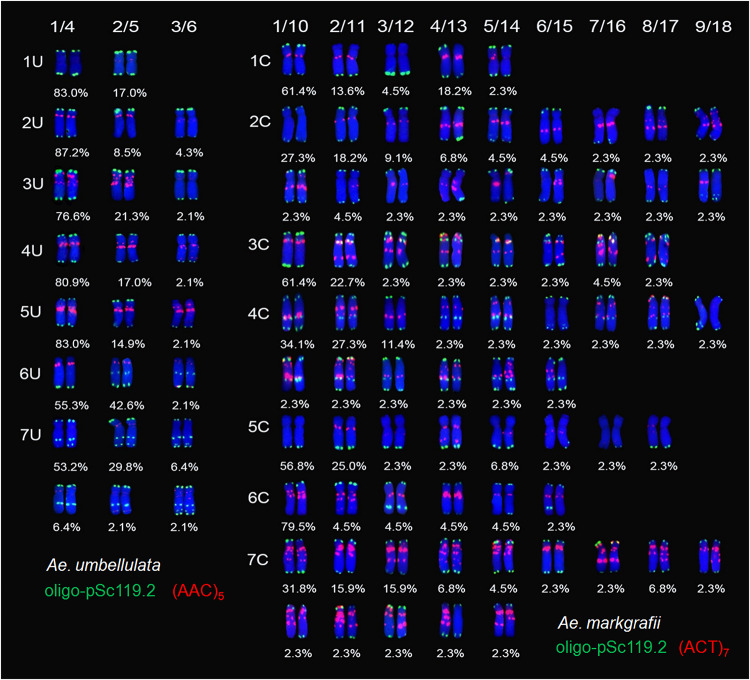
Chromosome variants and frequency analysis of *Ae. umbellulata* and *Ae. markgrafii* with the FISH probes oligo-pSc119.2/(AAC)_5_ and oligo-pSc119.2/(ACT)_7__._ Numbers indicate variants (numbered consecutively); in case these variants are shown in two rows (e.g., 7U, 2C), the number before the slash refers to the variant shown in the upper row, the number after the slash refers to the variant shown in the lower row.

All *Ae. umbellulata* accessions showed diverse hybridization signals for probes oligo-pSc119.2 and (AAC)_5_ and only one signal pattern for the oligo-pTa71 probe (Table 1 and Figure 2). The number of polymorphic variants for every U chromosome ranged from two to six (Figure 2).

Chromosome 1U had two variants for probe (AAC)_5_ and only one signal pattern for oligo-pSc119.2. Variant 1 has no (AAC)_5_ signal on the entire 1U chromosome, while variant 2 showed weak signals on the short arm (Figure 2). Each of the chromosomes 2U, 3U, 4U, 5U, and 6U had three variants for oligo-pSc119.2 and (AAC)_5_. The hybridization signals of the oligo-pSc119.2 probe on chromosomes 2U and 3U occurred on the telomeric regions of both the long and short arms (for variants 1 and 3) or the short arms (for variant 2). The 3U (AAC)_5_ hybridization signals varied in intensity (variants 1 and 2 vs. 3: strong vs. weak signal), whereas those of 2U varied in both the intensity and location (strong signal on central 2US for variants 1 and 2 and weak signal on the near-centromeric regions of 2US for variant 3). Among the three 4U variants, a major difference occurred for (AAC)_5_ rather than oligo-pSc119.2. All three 4U variants shared similar (AAC)_5_ signal sites on the pericentromeric regions and dissimilar signals on the short or long arm. Compared to 4U variant 1, variants 2 and 3 had an additional pair of signals on either the short or long arm. Chromosome 5U had three variants varying for the hybridization sites of both the oligo-pSc119.2 and (AAC)_5_ probes. Variant 3 lacked a pair of oligo-pSc119.2 signals that were specific to telomeric region of the short arm of variants 1 and 2. All three 5U variants shared strong (AAC)_5_ signals on the pericentromeric regions. Additionally, 5U variants 2 and 3 had an additional pair of signals on the short arms in comparison with variant 1. For the three 6U variants, variants 2 and 3 had a pair of oligo-pSc119.2 hybridization signals on the pericentromeric regions that were absent in variant 1. On the other side, variant 3 had an extra (AAC)_5_ telomeric signal on the long arms as compared to variants 1 and 2. Chromosome 7U had six variants. These 7U variants exhibited different hybridization sites for both the oligo-pSc119.2 and (AAC)_5_ probes. For example, an additional pair of oligo-pSc119.2 signals on the telomeric regions of the long arms was unique to variants 3 and 6, and a pair of oligo-pSc119.2 signals on the telomeric regions of the long arms was lacking in variant 4 in comparison with the other variants. The (AAC)_5_ signals of variants 2, 4, 5, and 6 were weak and appeared on the pericentromeric regions. Furthermore, weak (AAC)_5_ signals were also present on the telomeric regions of the long arms for variant 5.

A total of 25 FISH banding patterns were identified for the 47 *Ae. umbellulata* accessions (Table 1), of which the pattern harbored by PI 542367 (1, 1, 1, 1, 1, 2, 1) was the dominant variant (9 accessions, 19.1%). Seventeen of the 24 patterns were rare, with one accession for each of them.

#### Ae. markgrafii

All the *Ae. markgrafii* accessions showed polymorphic variants for probes oligo-pSc119.2 and (ACT)_7_ and identical signals for oligo-pTa71. The polymorphic variants for each C chromosome varied from 5 to 18 (Figure 2). Interestingly, heteromorphismin the homologous chromosomes was detected for every C chromosome.

Chromosome 1C had five variants, but only three variants (variants 1–3) showed identical signals between homologous chromosomes. Variants 2, 3, and 4 lacked a pair of oligo-pSc119.2 hybridization signals on the telomeric regions of the short arms in two (variants 2 and 3) or one (variant 4) 1C chromosome in comparison with the other two variants. Similarly, three of the five (except for variant 5 in one of the two 1C and variant 3) 1C variants showed (ACT)_7_ signal sites on the pericentromeric regions. A total of 18 variants for chromosome 2C were found, of which nine variants (variants 10–18) showed heteromorphismin the homologous chromosomes. All of the 2C variants showed oligo-pSc119.2 hybridization signals on the telomeric regions of both the long and short arms, except for variants 7 and 17, which lacked hybridization signals on the telomeric regions of the short arms. The telomeric signals of oligo-pSc119.2 on chromosome arm 2CL showed high diversity and could be arranged in four groups (I–IV; variants showing similar signal patterns were defined as a group, and the same meaning applies here after for groups), consisting of nine (variants 1, 2, 4, 7, 9, 11, 15, 16, and 17), three (variants 3, 6, and 8), three (variants 12, 13, and 14), and three variants (variants 5, 10, and 18). Group I had a pair of telomeric signals that was absent from group II. Both groups III and IV showed signals on one of the two 2C chromosomes; however, extra signal sites occurred on one of the two 2C chromosomes for group IV. All 18 variants except for variant 1 showed (ACT)_7_ hybridization signals on chromosome 2C. Heteromorphism in the homologous chromosomes 2C mainly occurred on the long arm. Chromosome 3C had eight variants, of which five variants (variants 1–5) shared similar (ACT)_7_ signals between homologous chromosomes. Seven of the eight 3C variants (except for variant 5) shared similar oligo-pSc119.2 signals. On chromosome arm 3CS, variant 5 had a pair of oligo-pSc119.2 signals, while the other seven variants had two pairs of signals. The eight variants also exhibited different (ACT)_7_ hybridization signals. For example, the (ACT)_7_ signals of variant 1 were located on the short arms, whereas an additional pair of signals was present on the long arms of variants 2, 3, and 5, and two further pairs of signals were present on both the long and short arms of variant 4. Chromosome 4C had 15 variants, but only eight variants (variants 1–8) showed consistent signals between homologous chromosomes. The oligo-pSc119.2 signal patterns of chromosome 4C could be divided into six groups, consisting of seven (variants 1, 2, 4, 5, 8, 14, and 15), two (variants 7 and 10), two (variants 6 and 9), two (variants 11 and 13), one (variant 3), and one (variant 12) variants. Group I had three pairs of signals on the telomeric regions on both the long and short arms and the central region of the long arms, while group II lost the signal on the telomeric region of the long arms. Group III showed three pairs of signals on the telomeric regions of both the long and short arms and proximal telomeric regions of the long arms. Group IV lost signals on the telomeric region of the long arms in one of the two 4C chromosomes when compared with group I. Group V had two pairs of signals on the telomeric regions of both chromosome arms, while group VI had an additional signal site on one of the two 4C chromosomes. Of the 15 4C variants, 14 variants (except for variant 6) showed (ACT)_7_ hybridization signals in the middle of the chromosome and extended from the long arm to the short arm. Chromosome 5C had eight variants. Of them, four variants (variants 1–4) shared similar hybridization signal sites between homologous chromosomes. All of the eight variants had a pair of oligo-pSc119.2 signals on the telomeric region of the long arms, except for variants 6 and 7, which lacked such hybridization signals on one of the two 5C. Six of the eight 5C variants (except for variants 1 and 7) had (ACT)_7_ signals distributed in different positions. Chromosome 6C had six variants, of which two variants (variants 5 and 6) showed heteromorphism in the homologous chromosomes. All of the six variants shared similar oligo-pSc119.2 signals on the telomeric region of the short arms, while an additional pair of oligo-pSc119.2 signals was present on the long arms of two 6C for variants 3 and 4, and one 6C for variants 5 and 6. All of the six variants had (ACT)_7_ signals spread around the pericentromeric regions. Chromosome 7C had 14 variants. Of them, seven variants (variants 8–14) exhibited heteromorphism in the homologous chromosomes. All of the 14 variants showed oligo-pSc119.2 signals on the telomeric regions of both chromosome arms, except for variants 10 and 14, which lacked a pair of signals on the telomeric regions of the long arms of one 7C for the former and double 7C for the latter. All of the 14 variants showed (ACT)_7_ signals mainly distributed on the pericentromeric regions of the long arms, except for one 7C in variant 13 that had three pairs of (ACT)_7_ signals fewer.

The 44 *Ae. markgrafii* accessions showed 43 different chromosome variants for the seven pairs of chromosomes (Table 1), suggesting that abundant FISH diversity had occurred within this species.

#### Ae. comosa

The 48 *Ae. comosa* accessions showed different signal patterns for the seven chromosomes as revealed by probes oligo-pSc119.2, (CTT)_12_, and oligo-pTa71 (Table 1). Biosystematically, *Ae. comosa* contains two subspecies, subsp. *comosa* and subsp. *subventricosa*. The main results of each subspecies were as follows.

##### Subspecies *comosa*

**FIGURE 3:**
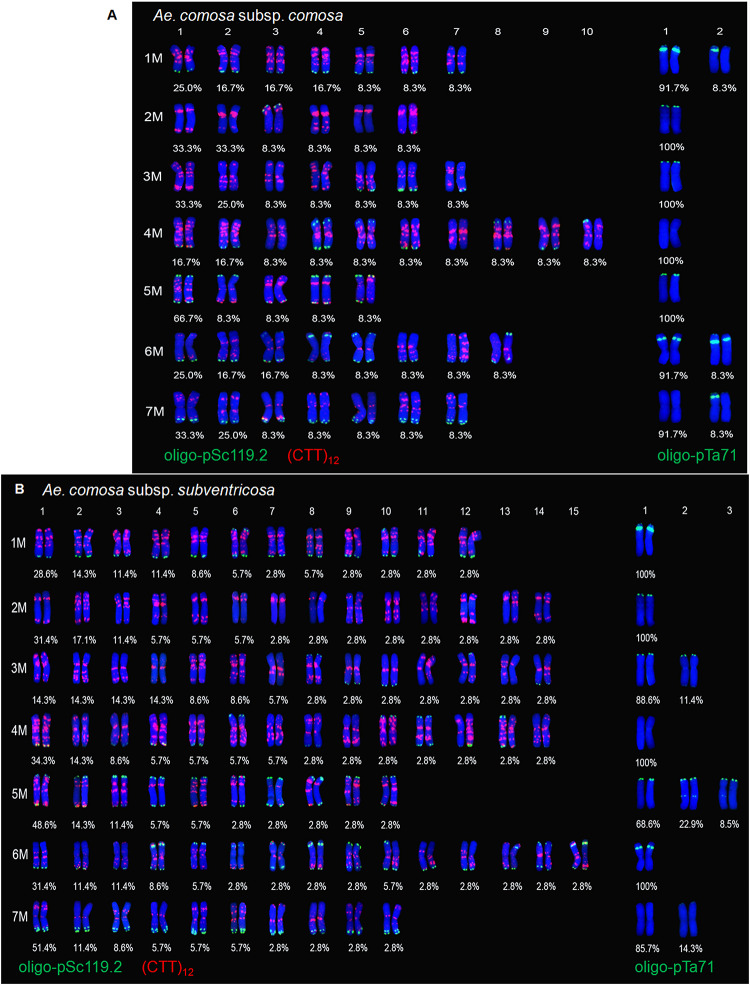
Chromosome variants and frequency analysis of *Ae. comosa* subspecies *comosa* **(A)** and *subventricosa* **(B)** with FISH probes oligo-pSc119.2, (CTT)_12_, and oligo-pTa71. Numbering of variants as explained for Figure 2.

The 13 accessions of subsp. *comosa* showed 5–10 variants for probes oligo-pSc119.2 and (CTT)_12_ in every M chromosome and two variants for probe oligo-pTa71 for each of three M chromosomes (1M, 6M, and 7M) (Figure 3A).

Chromosome 1M had seven variants for probes oligo-pSc119.2 and (CTT)_12_ and two variants for probe oligo-pTa71. The hybridization signals of oligo-pSc119.2 were located on the telomeric region of the long arms, while those of the (CTT)_12_ were distributed on the entire 1M chromosome (variants 2, 3, 4, and 6) or only on the pericentromeric regions (variants 1, 5, and 7). The 1M variant 2 of probe oligo-pTa71 lacked a pair of intensity signals on the subtelomeric regions of the short arms in one of the two chromosomes when compared with variant 1. Chromosome 2M had six variants for probes oligo-pSc119.2 and (CTT)_12_. Four variants (variants 1, 2, 4, and 5) lacked the oligo-pSc119.2 signal on both the long and short arms, while the other two variants had a pair of oligo-pSc119.2 signals on the short arm of two 2M (variant 3) and on the long arm of one 2M (variant 6). The hybridization signals of (CTT)_12_ were mainly distributed on the pericentromeric and telomeric regions of the long arms (such as variant 1) or only on the pericentromeric regions (such as variant 5). Chromosome 3M had seven variants for probes oligo-pSc119.2 and (CTT)_12_. Four variants (variants 1–4) lacked the oligo-pSc119.2 signal on the entire chromosome, while variants 5 and 6 had a pair of signals on the long and short arms, respectively. Variant 7 showed heteromorphism between two 3M homologous chromosomes for both the oligo-pSc119.2 and (CTT)_12_ signals. The (CTT)_12_ signals of chromosome 3M could be divided into three groups according to their signals on the short arm and pericentromeric regions (variants 2 and 3), on the long arm and pericentromeric regions (variants 5, 6, and 7), or on the whole chromosomes (variants 1 and 4). Chromosome 4M had 10 variants for probes oligo-pSc119.2 and (CTT)_12_, and the telomeric signals of probe oligo-pSc119.2 could be divided into five groups (I–V). All of the variants in groups I (variants 1, 2, 6, and 9) and II (variant 5) had a pair of telomeric signals located on the long and short arms, respectively. Two variants (variants 3 and 7) belonged to group III and lacked the oligo-pSc119.2 signal on both 4M chromosomes, and the variant in group V (type 10) lacked such a signal on one 4M. Two variants (variants 4 and 8) in group IV had a pair of subtelomeric signals on both the long and short arms. The (CTT)_12_ signals of chromosome 4M could be divided into two groups (I and II). The signals of the seven variants (variants 1, 2, 5, 6, and 8–10) in group I were distributed on the entire chromosome, while those of the three variants in groups II (variants 3, 4, and 7) were on the pericentromeric regions. Chromosome 5M had five variants for probes oligo-pSc119.2 and (CTT)_12_. Variants 1 and 4 had a pair of telomeric signals for the oligo-pSc119.2 probe on both the long and short arms, while variants 2 and 3 had a pair of oligo-pSc119.2 signals on either the short or long arms. Variant 5 showed heteromorphism between two 5M homologous chromosomes, with one 5M lacking a pair of oligo-pSc119.2 signals. The (CTT)_12_ signals were distributed on the entire chromosomes (variant 1), on the long arms (variant 2), or on both the long and short arms (variants 3, 4, and 5). Chromosome 6M had eight variants for probes oligo-pSc119.2 and (CTT)_12_, and the hybridization signals of oligo-pSc119.2 could be divided into three groups (I–III). The variants among the three groups showed difference in a pair of oligo-pSc119.2 signals. Three variants (variants 3, 4, and 5) in group I and four variants (variants 1, 2, 6, and 7) in group II had signals on both chromosome arms and only on the long arms, respectively, while the sole variant (variant 8) in group III lacked a pair of signals on one of the two 6M chromosomes. The (CTT)_12_ signals were located on the short arm and pericentromeric regions (variants 2, 4, 6, and7), on the long arms and pericentromeric regions (variants 3 and 5), or on the long and short arms (variant 1). Chromosome 6M had two variants for probe oligo-pTa71. Both variants 1 and 2 shared strong oligo-pTa71 signals on the nucleolar organizer regions, whereas variant 2 had an additional pair of oligo-pTa71 signals on the telomeric regions of the short arm on one of the two 6M chromosomes. Chromosome 7M had seven variants for probes oligo-pSc119.2 and (CTT)_12_ and two variants for probe oligo-pTa71. All of these variants shared two pairs of oligo-pSc119.2 telomeric signals on the long arms, except for variant 2, which had lost a pair of such signals. The (CTT)_12_ signals were located on the short arm and pericentromeric regions (variants 2, 4, 6, and 7), on the long arms and pericentromeric regions (variants 3 and 5), or on the long and short arms (variant 1). The 7M variant 2 of probe pTa71 differed from variant 1 by the existence of a pair of weak signals on the telomeric regions of both 7M and a pair of strong signals on the subtelomeric region of one of the two 7M chromosomes.

##### Subspecies *subventricosa*

The 35 accessions of subspecies *subventricosa* showed 10–15 variants for probes oligo-pSc119.2 and (CTT)_12_ in all of the seven M chromosomes, 2–3 variants in three (3M, 5M, and 7M) of seven chromosomes, and only one signal pattern for the remaining four chromosomes for probe oligo-pTa71 (Figure 3B).

Chromosome 1M had 12 variants for probes oligo-pSc119.2 and (CTT)_12_ and two variants for probe oligo-pTa71. All of the variants shared oligo-pSc119.2 signals on the telomeric regions, but the signals of most variants (10 variants) were located on the long arms, while those of the remaining two variants (variant 6 and one of the two 1M in variant 10) were on the short arms. The (CTT)_12_ signals of 1M could be divided into four groups (I–IV), comprising seven (variants 2, 4, 6, 7, 8, 9, and 12), one (variant 5), two (variants 3 and 10), and two (variants 1 and 11) variants, respectively. The hybridization signals of groups I, II, III, and IV were distributed on the entire 1M chromosome, on the pericentromeric regions, on both the long and short arms and pericentromeric regions, and on the pericentromeric regions and the short arms, respectively. Both variants 1 and 2 shared pTa71 signals on the telomeric regions of the short arms, but such a signal was lacking in one of the 1M homologous chromosomes of variant 2. Chromosome 2M had 14 variants for probes oligo-pSc119.2 and (CTT)_12_ and only one signal pattern for probe oligo-pTa71. All of these variants lacked the oligo-pSc119.2 signal, but their hybridization signals of (CTT)_12_ showed obvious differences, as they were mainly distributed on the pericentromeric regions and the long arms (such as variant 1) or only on the pericentromeric regions (such as variants 6 and 7). Chromosome 3M had 14 variants for probes oligo-pSc119.2 and (CTT)_12_ and two variants for probe oligo-pTa71. The oligo-pSc119.2 signals of chromosome 3M could be classified into three groups (I–III). Nine variants (variants 1–7, 11, and 12) in group I lacked the oligo-pSc119.2 signal on the entire chromosome. Four variants (variants 8, 9, 10, and 14) in group II had a pair of signals on the long or short arms, and the sole variant (variant 12) in group III had signals on one of the two 3M chromosomes. The (CTT)_12_ signals of this chromosome could be divided into four groups based on the distribution of the signals on the short arms and pericentromeric regions (variants 7 and 11), on the long arms and pericentromeric regions (variants 2, 3, 5, 8, and 12), on the pericentromeric regions (variants 4,9, 10, 13 and 14), or on the entire chromosomes (variants 1 and 6). Both the two variants of the pTa71 probe shared telomeric signals on the short arms, while variant 2 had two additional pairs of signals on the near-centromeres and long arms. Chromosome 4M had 14 variants for probes oligo-pSc119.2 and (CTT)_12_ and only one signal pattern for probe oligo-pTa71. The signal pattern of probe oligo-pSc119.2 could be classified into five groups (I–V). Seven variants (variants 1, 3, 4, 5, 8, 12, and 14) in group I and three variants (variants 6, 9, and 10) in group II had a pair of telomeric signals on the long and short arms, respectively. The two variants (variants 2 and 7) in group III lacked the oligo-pSc119.2 signal. The sole variant in group IV (variant 11) had a pair of subtelomeric signals on both the long and short arms, and the only variant (variant 13) in group V did not possess a pair of oligo-pSc119.2 signals on one of the two 4M chromosomes. Moreover, the (CTT)_12_ signals of chromosome 4M could be divided into three groups (I–III). The signals of the seven variants (variants 1, 2, 4, 5, 10, 12, and 13) in group I were distributed on the entire chromosome, while those of the two variants each in group II (variants 3, 6, 7, 9, 11, and 14) and the sole variant (variant 8) in group III had lacked the (CTT)_12_ signal on the short and long arms, respectively. Chromosome 5M had 10 variants for probes oligo-pSc119.2 and (CTT)_12_ and three variants for probe oligo-pTa71. The 10 variants had different (CTT)_12_ signals that were located on the entire chromosome (variants 1, 5, and 9), on the short arms (variant 4), on the pericentromeric regions and long arms (variant 3, 7, 8, and 10), or on both the long and short arms (variants 2 and 6). Chromosome 5M had three variants for probe oligo-pTa71. Variant 1 had a pair of oligo-pTa71 signals on the telomeric regions of the short arms, and variant 2 had a pair of signals on the middle of the long arms and on the telomeric regions of the short arms. One of the two 5M chromosomes in variant 3 had lost a pair of oligo-pTa71 signals that were located in the middle of the long arms in variant 2. Chromosome 6M had 15 variants for probes oligo-pSc119.2 and (CTT)_12_ and only one variant for probe oligo-pTa71. The hybridization signals of probe oligo-pSc119.2 could be divided into three groups (I–IV). Seven variants (variants 4, 6–9, 14, and 15) in group I, five variants (types 1–3, 11, and 12) in group II, and the sole variant (variant 5) in group III had a pair of signals located on both the long and short arms, on the long arms, and on the short arms, respectively. Two variants (types 10 and 13) in group IV lacked a pair of signals on one of the two 6M homologous chromosomes. The (CTT)_12_ signals of these variants were distributed on entire chromosomes (variants 4, 5, 13, and 15), the short arms and pericentromeric regions (variant 7), the long arms and pericentromeric regions (variants 1–3, 9–12, and 14), and both chromosome arms (variants 6 and 8). Chromosome 7M had 10 variants for probes oligo-pSc119.2 and (CTT)_12_ and three variants for probe oligo-pTa71. These variants had different (CTT)_12_ signals that were located on the short arm and pericentromeric regions (variants 3, 5 and 7), on the long arms and pericentromeric regions (variants 4 and 8), on the pericentromeric regions (variant 2), or on the entire chromosomes (variants 1, 6, 9, and 10). Chromosome 7M had three variants for probe oligo-pTa71, of which variant 1 lacked such a signal, while variants 2 and 3 shared a pair of telomeric signals on the short arms, and variant 3 had an additional pair of strong oligo-pTa71 signals on the subtelomeric regions in one of the two 7M chromosomes.

#### Ae. uniaristata

**FIGURE 4:**
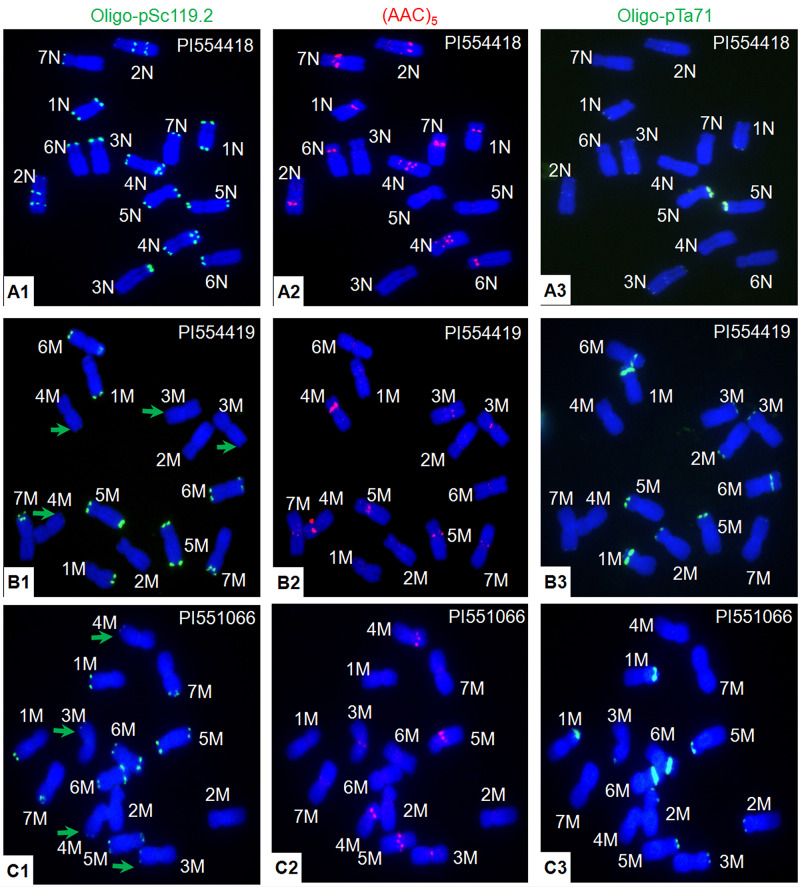
Comparison of the hybridization patterns of probes oligo-pSc119.2 **(A1–C1)**, (AAC)_5_ **(A2–C2)**, and oligo-pTa71 **(A3–C3)** on the metaphase chromosomes of *Ae. uniaristata* PI 554418 **(A1–A3)**, PI 554419 (formerly *Ae. uniaristata*, now *Ae. comosa*,**B1–B3**), and *Ae. comosa* PI 551066 **(C1–C3)**. The different hybridization signals of oligo-pSc119.2 between PI 554419 and PI 551066 on 3M and 4M are indicated by green arrowheads.

Fluorescence *in situ* hybridization karyotyping of the six *Ae. uniaristata* accessions was conducted with probes oligo-pSc119.2, (AAC)_5_, and oligo-pTa71, but no variant was found (Figures 1, 4). Using PI 554418 as an example (Figures 4A1–A3), the hybridization signals of oligo-pSc119.2 were located on the telomeric regions of chromosome arms 2NS, 3NS, 6NS, and 7NS and chromosomes 1N, 2N, and 4N. Additionally, a pair of oligo-pSc119.2 signals was also present in the middle of chromosome arm 2NL and the subtelomeric region of chromosome arm 4NL. The hybridization signals of probe (AAC)_5_ existed on five (except for 3N and 5N) of the seven N chromosomes. The signals of chromosome 4N were distributed on the pericentromeric regions, middle of the short arms, and near the pericentromeric regions of the long arms, and those of 1N and 7N, and 6N were located on the pericentromeric regions and the middle of the short arms, respectively.

### Comparison of the FISH Pattern of PI 554419 in *Ae. uniaristata* and *Ae. comosa*

Previously, PI554419 was classified as *Ae. uniaristata*^1^. Many differences were detected between the FISH karyotypes of PI 554419 and *Ae. uniaristata* PI554418; however, similar FISH karyotypes were found between PI 554419 (Figures 4B1–B3) and *Ae. comosa* PI 551066 (Figures 4C1–C3) as revealed by probes oligo-pSc119.2, (AAC)_5_, and oligo-pTa71. Thus, based on the FISH karyotypes, PI 554419 should be treated as *Ae. comosa*, although a minor difference was detected between PI 554419 and PI 551066. For example, PI 554419 had no weak telomeric signals on chromosome arms 3ML and 4ML in comparison with PI 551066. Furthermore, PI554419 was verified as *Ae. comosa* by comparison with the herbarium specimens of both species^2^.

### Heteromorphism in the Homologous Chromosomes of *Ae. markgrafii* and *Ae. comosa*

Some *Ae. markgrafii* and *Ae. comosa* accessions showed heteromorphism in the homologous chromosomes (*viz.*, two 1M or two 1C, etc.).

**FIGURE 5:**
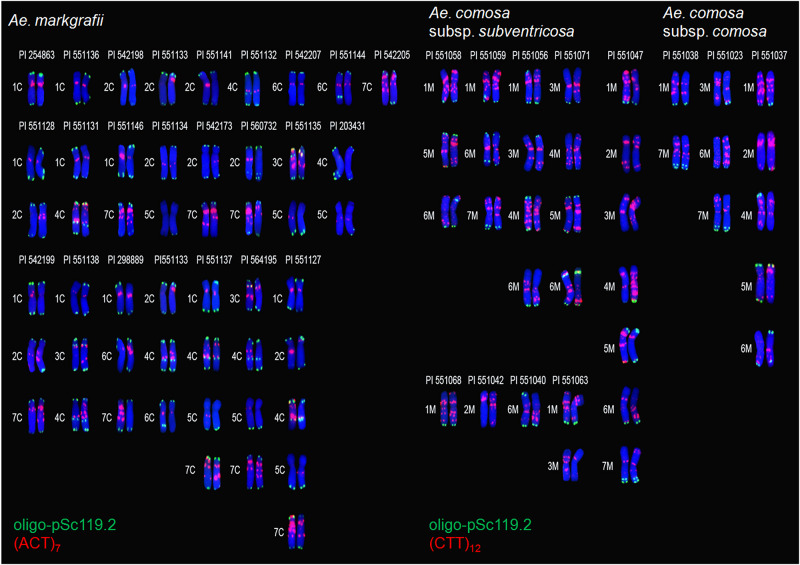
Heteromorphic FISH hybridization signals between the homologous chromosomes of *Ae. markgrafii* (left) and *Ae. comosa* (right).

**FIGURE 6:**
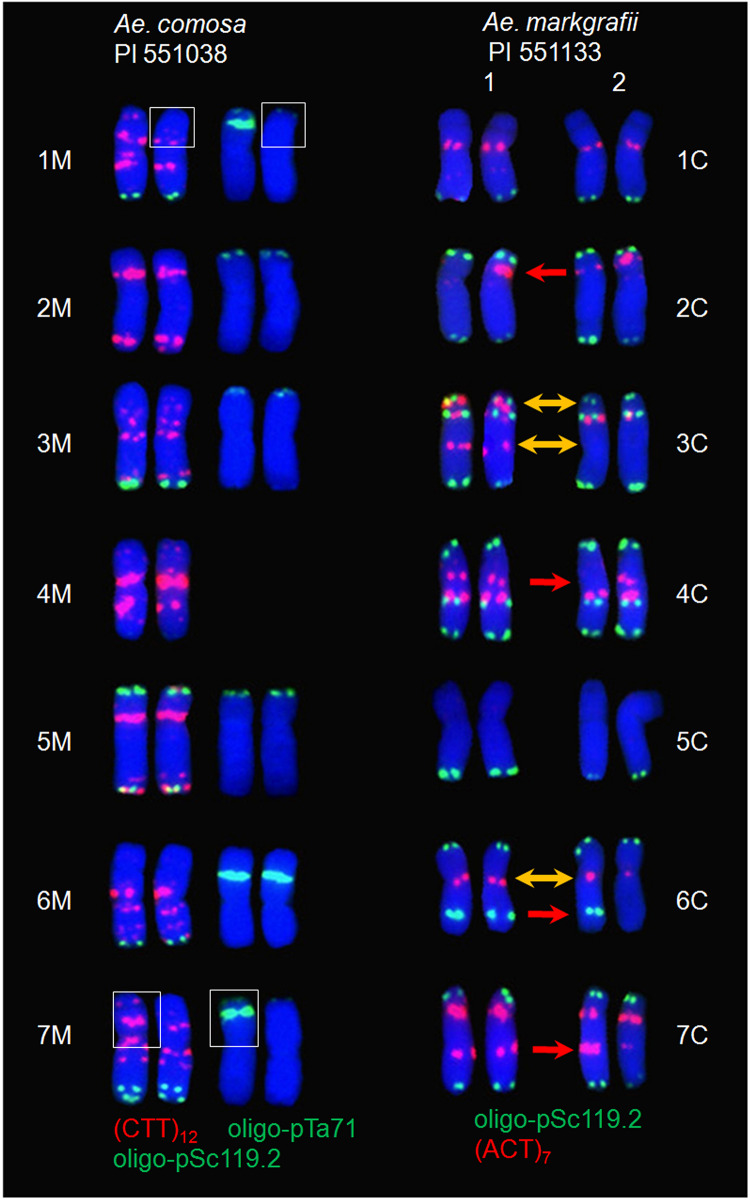
FISH karyotyping of *Ae. comosa* (PI 551038) and *Ae. markgrafii* (PI 551133) using DNA probes. The translocation between the chromosome arms 1MS and 7MS of *Ae. comosa* PI 551038 is shown by white boxes. Two types of reproducible FISH signal patterns were observed within the same metaphase cells of *Ae. markgrafii* PI551133 at a ratio of 1:1. Signal differences of the same and different types between homologous chromosomes are indicated by red and yellow arrowheads, respectively.

A total of 24 *Ae. markgrafii* accessions showed heteromorphism in the homologous chromosomes for probes oligo-pSc119.2 and/or (ACT)_7_, of which nine, eight, four, two, and one accessions exhibited heteromorphism between one to five, respectively, pairs of homologous chromosomes (Figure 5). Some accessions exhibited heteromorphism between a pair of homologous chromosomes in each of the five C chromosomes (1C, 2C, 4C, 6C, and 7C). For example, PI 254863 and PI 551136 showed heteromorphic oligo-pSc119.2 signals between two 1C homologous chromosomes. Six of the seven C chromosomes (all except for 6C) exhibited seven types of heteromorphism [1C with 2C (PI 551128), 4C (PI 551131), and 7C (PI 551146); 2C with 5C (PI 551134) and 7C (PI 542173 and PI 560732); as well as 5C with 3C (PI 551135) and 4C (PI 203431)] between two pairs of homologous chromosomes. Similarly, six of the seven chromosomes (all except for 5C) participated in the formation of three types of heteromorphism [1C with 2C and 7C (PI 542199), 3C and 4C (PI 551138), and 6C and 7C (PI 298889)] among three pairs of homologous chromosomes. Two accessions showed heteromorphism among four pairs of homologous chromosomes [4C, 5C, and 7C with 1C (PI 551137), and 3C (PI 564195)]. Two types of signal patterns were found in different cell division phases from the same root tips of PI551133 at a ratio of nearly 1:1 (Figure 6), which exhibited inconsistent signals between each of the five pairs of chromosomes 2C, 3C, 4C, 6C, and 7C. Chromosome 2C type 1 lacked a pair of (ACT)_7_ signals on the short arm in one of the two chromosomes as compared to type 2. On the contrary, chromosome 4C type 2 lacked a pair of (ACT)_7_ signals located on the long arm in one of the two chromosomes 4C in type 1. Similarly, chromosome 6C type 2 lacked a pair of oligo-pSc119.2 signals and a single (ACT)_7_ signal located on the long arms in one of the two chromosomes 6C and on the pericentromeric regions of two 6C in type 1. Chromosome 3C (type 2) lacked two pairs of (ACT)_7_ signals on the pericentromeric regions and telomeric regions of the short arm compared to type 1. Chromosome 7C type 2 lacked a single (ACT)_7_ signal on the near-centromeric regions in one of the two chromosomes as compared to type 1. PI 551127 exhibited heteromorphism among five pairs (1C, 2C, 4C, 5C, and 7C) of homologous chromosomes (Figure 5).

A total of 12 *Ae. comosa* accessions (three accessions of subsp. *comosa* and nine of subsp. *subventricosa*) showed heteromorphic oligo-pSc119.2 and/or (CCT)_12_ signals between homologous chromosomes, of which three, two, three, two, one, and one accessions showed heteromorphism between one, two, three, four, five and seven, respectively, pairs of homologous chromosomes, respectively (Figure 5). Three M chromosomes (1M, 2M, and 6M) exhibited heteromorphism between a pair of homologous chromosomes. For example, PI 551068, PI 551142, and PI 551140 (all belonged to subsp. *subventricosa*) showed heteromorphism between two 1M, 2M, and 6M, respectively. PI 551038 (subsp. *comosa*) also showed heteromorphism between two pairs of 1M and 7M in both the (CTT)_12_ and oligo-pTa71 signals. Further analysis suggested that a translocation had occurred on the short arms between one of the two chromosome arms 1MS and 7MS (Figure 6). Three chromosomes (1M, 3M, and 7M) were involved in two types of heteromorphism [1M with 3M (PI 551063 subsp. *subventricosa*) and 7M (PI 551038 subsp. *comosa*)] between two pairs of homologous chromosomes. Five chromosomes (1M, 3M, 5M, 6M, and 7M) exhibited three types of heteromorphism [6M with 1M and 5M (PI 551058), 1M and 7M (PI 551059) in subsp. *subventricosa*, and 3M and 7M (PI 551023) in subsp. *comosa*] among the three pairs of homologous chromosomes. Two accessions of subsp. *subventricosa* showed heteromorphism among the four pairs of homologous chromosomes [3M, 4M, and 6M with 1M (PI 551056), and 5M (PI 551071)]. One accession (PI 551037 subsp. *comosa*) exhibited heteromorphism among five pairs (1M, 2M, 4M, 5M, and 6M) of homologous chromosomes. Not expectedly, PI 551047 (subsp. *subventricosa*) showed heteromorphism for all of the seven pairs of homologous chromosomes.

## Discussion

Tandem repeats were previously considered to be junk DNA generated during evolution that lack any biological function (Doolittle and Sapienza, 1980). Now, it is generally accepted that tandem repeats play a pivotal role in chromosome organization, stabilization, and recombination as well as DNA replication (Heslop-Harrison, 2000; Li et al., 2002; Gemayel et al., 2012). Variation in the number and distribution of tandem repeat sequences may be involved in speciation (Flavell et al., 1979; Li et al., 2004; Britten, 2010) and were shown to accelerate the evolution of coding and regulatory sequences (Gemayel et al., 2010). Thus, the diversity of tandem repeat sequences can be used to assess genetic relatedness from the species level to the genome level (Dvorak and Zhang, 1992; Mehrotra and Goyal, 2014).

### Genetic Diversity Among Four Diploid *Aegilops* Species With U, C, M, and N Chromosomes

Fluorescence *in situ* hybridization karyotyping is a valid tool for chromosome authentication, species classification, and evolutionary studies (Badaeva et al., 2002, 2004). It is also reliable for identifying alien chromosome/fragment introgression in wheat-wild distant hybridization crosses when combined with genome *in situ* hybridization (Wang et al., 2016). A large number of polymorphic FISH karyotypes were detected among and within *Ae. umbellulata, Ae. markgrafii*, and *Ae. comosa*, whereas identical karyotypes were observed among *Ae. uniaristata* accessions due to the limited accessions that were used (Figures 2, 3).

Similar oligo-pSc119.2 signals were mainly presented on the telomeric regions of the long and short arms of the four *Aegilops* species with C, M, N, and U genomes, while different signals were detected either among species or among seven homologous (Figures 1–3). Polymorphic FISH signals of probe oligo-pSc119.2 were present on 2D, 3D, and 4D of *Ae. tauschii* (Zhao et al., 2018); 2U, 4U, 5U, and 6U of *Ae. umbellulata*; and 4M and 6M of *Ae. comosa* (Schneider et al., 2005). Additionally, polymorphic oligo-pSc 119.2/(AAC)_5_ signals were also discovered for *Ae. umbellulata* chromosomes 1U, 6U, and 7U (Song et al., 2019). For example, compared with previous studies, extra oligo-pSc119.2 signals were discovered on the telomeric regions of 7UL in *Ae. umbellulata* (Schneider et al., 2005), on the telomeric regions of 2CL and 6CL in *Ae. markgrafii* (Danilova et al., 2017), on the proximal telomeric and subtelomeric regions of 1ML, 3ML, and 3MS in *Ae. comosa*, as well as on the near-centromeres and near central position of 2N in *Ae. uniaristata* (Badaeva et al., 1996a; Schneider et al., 2005; Kwiatek et al., 2013). Furthermore, the oligo-pSc119.2 signal was absent on one of the chromosomes arms 5CL of *Ae. markgrafii*, 6MS of *Ae. comosa*, and 6NL of *Ae. uniaristata* (Badaeva et al., 1996a).

The 45S rDNA, a tandem repeat sequence that is located on the nucleolar organizer region of satellite and some non-satellite chromosomes with only a few copy numbers, is represented by the occurrence of the Oligo-pTa71 signals (Long and Dawid, 1980; Mukai et al., 1991; Tang et al., 2014). The pTa71 signals in the present study were mainly located on the telomeric regions of homologous 1 and 5 on the short arms, which is consistent with previous studies (Yamamoto, 1992; Badaeva et al., 1996b; Mirzaghaderi et al., 2014; Song et al., 2019). Additional weak pTa71 signals were present on the telomeric regions of chromosome arm 1NL, the pericentromeric regions of 2N and the telomeric regions of chromosome arm 2NS, and the pericentromeric regions and telomeric regions of chromosome 3N in *Ae. uniaristata*. Extra strong pTa71 signals were observed on the telomeric regions of chromosome arms 2MS, 3MS, 6MS, and 7MS and on the pericentromeric regions of chromosome 5M in *Ae. comosa*.

In addition to polymorphic signals revealed by the oligo-pSc119.2 and pTa71 probes, the microsatellite probes (AAC)_5_, (CTT)_12_, and (ACT)_7_ exhibited more hybridization sites on nearly all the chromosomes, and their signals were distributed mainly on the pericentromeric regions and extended to the whole chromosomes according to the probe used. Usually, the (CTT)_n_-based FISH patterns of some *Aegilops* species are very similar to their C-banding patterns (Ruban and Badaeva, 2018). The (CTT)_12_ signals of four diploid *Aegilops* species are consistent with their C-banding patterns (Figure 1; Friebe et al., 1992, 1995, 1996b), although minor differences occurred in some chromosomes including 1U, 5U, 3C, 6C, 2M, and 6M as well as other chromosomes. For example, the (CTT)_12_ signal of *Ae.comosa* showed extensive diversity both between two subspecies and among the seven chromosomes (Figure 3). The present results revealed abundant FISH variants both among and within species.

### Intra- and Interspecific Genetic Variations in FISH Patterns and Their Implication in Evolution and Speciation

Intra- and interspecific genetic diversity was detected in the FISH patterns. Slightly higher FISH polymorphisms of oligo-pSc119.2 signals were detected in *Ae. comosa* than *Ae. markgrafii*, and both were higher than *Ae. umbellulata*. Our FISH results are very similar to previous reports where *Ae. comosa* showed higher genetic diversity than *Ae. umbellulata* (Resta et al., 1996; Monte et al., 2001; Schneider et al., 2005), where intra- and interspecific genetic diversity was evaluated by using restriction fragment length polymorphism (RFLP), amplified fragment length polymorphism (AFLP), and FISH karyotypes. Similarly, a sequence-tagged site based on molecular markers suggested that the U genome of *Ae. umbellulata* showed less genetic polymorphism than the M genome of *Ae. comosa* and C genome of *Ae. markgrafii* (Chee et al., 1995). On the contrary, a low level of intraspecific variation was discovered with AFLP markers among seven diploid *Aegilops* species, including *Ae. umbellulata* and *Ae. markgrafii*, except for the cross-pollinating *Ae. speltoides* and *Ae. mutica* (Sasanuma et al., 2004).

Intraspecific genetic diversity was also found among the diploid *Aegilops* species with U, C, and M genomes as revealed by oligo-pSc119.2 in combination with a microsatellite probe (AAC)_5_, (ACT)_7_, and(CTT)_12_, respectively. More FISH signal patterns were present in each homologous chromosome of *Ae. comosa* (5–10 and 10–15 variants for subsp. *comosa* and subsp. *subventricosa*, respectively) and *Ae. markgrafii* (5–18 variants) than those of *Ae. umbellulata* (two to six variants), and *Ae. uniaristata* (one variant). The *Ae. umbellulata* 7U had greater genetic diversity, while 1U had few patterns among the seven U chromosomes (Figure 2). The number of polymorphic FISH patterns among the seven C chromosomes of *Ae. markgrafii* was ranked as 2C (18 variants) > 4C (15) > 7C (14) > 3C = 5C (8) > 6C (6) > 1C (5) (Figure 2). Meanwhile, the FISH patterns of *Ae. comosa* subsp. *comosa* in 13 accessions were richer than that of subsp. *subventricosa* in 35 accessions and were ranked as 4M (10 variants) > 6M (8) > 1M, 3M and 7M (7) > 2M (6) > 5M (5) and 6M (15 variants) > 2M, 3M and 4M (14) > 1M (12) > 5M and 7M (10) for each subspecies (Figure 3). Intraspecific variation between two subspecies of *Ae. comosa* was also discovered with the C-banding karyotype, and an obvious difference occurred mainly on the pericentromeric and nucleolar organizing regions (Teoh et al., 1983; Friebe et al., 1996a). Several polymorphic variations for C-banding size and position are present among *Ae. markgrafii* (Friebe et al., 1992, *Ae. umbellulata*, *Ae. uniaristata*, and *Ae. comosa* accessions (Friebe et al., 1995, 1996b). Considerable genetic diversity among *Ae. umbellulata* accessions has also been revealed by RNA sequencing analysis (Okada et al., 2018).

Alterations in the number and distribution of tandem repeats are one of the most important manifestations of genetic variation (Gemayel et al., 2010). Currently, abundant intra- and interspecific genetic variations in tandem repeats among four diploid *Aegilops* species were evaluated by the heterochromatin limited repetitive DNA probe pSc119.2, a tandem repeat sequence 45S rDNA-related DNA probe pTa71, and microsatellite sequence probes (AAC)_5_, (ACT)_7_, and (CTT)_12_. Previous studies have shown tandem repeat variations are involved in speciation and evolution as well as in phenotypic variation (Nagaki et al., 1998; Gemayel et al., 2010). For example, the copy numbers of tandem repeat Afa-family sequences per genome among Triticeae species are highly variable, suggesting that the amplification or deletion of such sequences is related to the evolution and speciation of Triticeae. In hexaploid wheat, the Afa-family sequences between the A and B genomes did not evolve in a concerted manner, and these sequences were amplified all over the chromosomes of the D-genome in a short period (Nagaki et al., 1998). The intraspecific variability of *Aegilops speltoides-*specific *Spelt1* and *Aegilops-Triticum*-specific *Spelt 52* tandem repeats in tetraploid and hexaploid wheat decreased sharply when compared with that of *Ae. speltoides*, with the exception of *Triticum timopheevii* Zhuk and *T. carthlicum* Nevski; both species maintain the amounts of Spelt1 unaltered because they are exceptional in being endemic species with restricted geographical distributions (Pestsova et al., 1998; Salina et al., 2006; Zoshchuk et al., 2009).

Moreover, the number and distribution of the tandem repeat sequences *Spelt1* and *Spelt 52* in *Ae. speltoides* exhibited a distinctive geographical gradient, with the number of *Spelt 1* in the central population of the species distribution being 12–14 times higher than in marginal populations. The changes in the number of these tandem repeats along an eco-geographical gradient may be ascribed to the depletion of tandem repeats in the marginal populations as a consequence of increased recombination rate under stressful conditions or the accumulation of tandem repeats in conducive climatic/edaphic environments in the central populations (Raskina et al., 2011).

### Heteromorphism in Homologous Chromosomes

Heteromorphism in homologous chromosomes has been identified in humans and plants using different cytogenetic methods (Suciu, 1986; Lapitan et al., 1988) and has also been detected in *Ae. comosa* and *Ae. markgrafii* as revealed by the FISH probes oligo-pSc119.2 and oligo-pTa71 plus a microsatellite probe (CTT)_12_ or (ACT)_7_ (Figures 2, 3, 5). All of the seven chromosomes of *Ae. comosa* and *Ae. markgrafii* displayed heteromorphism among one to seven or one to five pairs of homologous chromosomes in each species (Figures 5, 6). Heteromorphism was also discovered in four accessions each of *Ae. speltoides* as revealed by FISH probes oligo-pSc119.2, between four pairs of homologous chromosomes (1S, 4S, 5S, and 6S) and pAesp_SAT86, and between all seven pairs of homologous chromosomes (Dong et al., 2017; Ruban and Badaeva, 2018). In addition to the heteromorphic FISH signals between homologous pairs, heteromorphic C-banding patterns between two homologous chromosomes of B and D were also reported in one *Ae. markgrafii* accession (Friebe et al., 1992) and also occurred between the homologous chromosomes of 1R (Alkhimova et al., 1999), and 3R, 4R, 6R, and 7R (Lapitan et al., 1988) of rye (*Secale cereale*).

Heteromorphism in homologous chromosomes is one manifestation of genetic variation. These genetic variations may have resulted from alterations in chromosome structures, such as chromosome rearrangements, translocations, and inversion between chromosomes within these accessions (Friebe et al., 1992, 1995, 1996b; Badaeva et al., 2004; Schneider et al., 2005). Moreover, the frequent occurrence of heteromorphic chromosomes could be an indicator of open pollination. In Triticeae, *Ae. speltoides* and rye (*Secale cereale*) are typical outcrossers with heteromorphism in homologous chromosomes (Lapitan et al., 1988; Ruban and Badaeva, 2018), while *Ae. markgrafii* is a facultative cross-pollinating plant with a highly asymmetrical karyotype that is indicative of chromosome rearrangements (Kilian et al., 2011; Danilova et al., 2017). Different from *Ae. markgrafii*, *Ae. comosa* is a self-pollinating plant (Friebe et al., 1996a). We speculate that the high level of heteromorphic FISH patterns observed in *Ae. markgrafii* and *Ae. comosa* is the consequence of hybrid karyotypes, which may be caused by outcrossing within genotypes and/or by chromosomal rearrangements (Tang et al., 2011; Danilova et al., 2017).

In addition to the heteromorphism in homologous chromosomes of FISH karyotypes within *Ae. markgrafii* and *Ae. comosa* (Figure 5), a translocation between the 1MS and 7MS of *Ae. comosa* PI 551038 and two types of FISH signals at a ratio of 1:1 within the same root cells of *Ae. markgrafii* PI 551133 were another form of genetic variation (Figure 6). A translocation on the 4S of *Ae. speltoides* may be ascribed to a center inversion, which was detected by the probes (CTT)_10_ and pAesp_SAT86 (Ruban and Badaeva, 2018). Although so many genetic variations were identified, only a few translocations were identified, which may be because the FISH probes were too small. These variations may have originated from chromosomal rearrangements during the evolutionary process, and this recombination affects the synteny between the homologous chromosomes of *Aegilops* and *Triticum* (Devos et al., 1993; Zhang et al., 1998).

## Conclusion

Chromosome structural variations of four diploid *Aegilops* species were evaluated by FISH karyotypes. Extensive intra- and interspecific genetic variation was found in *Ae. umbellulata, Ae. markgrafii*, and both subspecies of *Ae. comosa*, but not in *Ae. uniaristata*. In both subspecies of *Ae. comosa* and in *Ae. markgrafii*, frequently occurring heteromorphism in homologous chromosomes constituted an additional component of chromosomal variation. These results will provide important clues for understanding chromosome organization and evolutionary relationships as well as speciation among *Aegilops* species.

## Data Availability Statement

The datasets generated for this study are available on request to the corresponding author.

## Author Contributions

ZS conducted the experiments and drafted the manuscript. TB, YZ, and QX analyzed the data. JL and GL prepared the plant materials. SD and ZY conceived and designed the study. ZY revised the manuscript.

## Conflict of Interest

The authors declare that the research was conducted in the absence of any commercial or financial relationships that could be construed as a potential conflict of interest.

## Footnotes

1 http://www. ars -grin.gov/

2 http://www.cvh.ac.cn/her/SAUT
